# Amassing Efforts against Alien Invasive Species in Europe

**DOI:** 10.1371/journal.pbio.0040279

**Published:** 2006-08-15

**Authors:** Susan M Shirley, Salit Kark

Almost daily, we can read newspaper articles voicing concerns about alien species and their impacts on native biodiversity, economic resources, and human health. Alien or non-native species introductions by humans rank as one of the two top factors (after habitat loss) leading to declines in biological diversity [1]. Their synergistic effects with changes in land-use and climate may lead to even more severe declines in the future [2]. Globally, there is a sense of urgency for practical steps to be taken to strictly identify and control the introduction of alien species and manage species that have already become invasive [3,4].

Although scientists and policymakers are becoming increasingly aware that introductions of alien species impose serious impacts [3,5], there are large differences in how nations deal with the issue. While some countries have detailed lists of alien species and well-established protocols for their trade and control, information in other countries is almost non-existent. Once established in a small area, species can enlarge their range across wide geographical areas, sometimes rapidly, so tackling the problem requires a strategic approach involving cooperation of many countries. Efforts to come to grips with this problem led to The Global Invasive Species Programme (GISP) [6].

## Integrating Information across Europe

**Figure 1:**
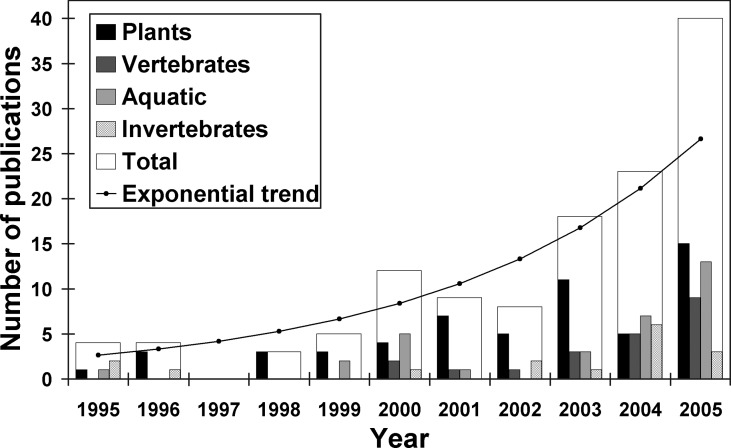
Change in the Number of Publications on Alien Species in Europe That Appeared in Eight Major Ecology and Conservation Biology Journals from 1995–2005 An increase with year was found for the plant, vertebrate, and aquatic taxonomic groups (*R^2^* = 0.81 for all four groups combined; plants: linear *R^2^* = 0.67, *p* = 0.002; vertebrates: linear *R^2^* = 0.67, *p* = 0.002; aquatic: linear *R^2^* = 0.51, *p* = 0.014; invertebrates: linear *R^2^* = 0.28, *p* = 0.091). The journals include *Biological Conservation, Biological Invasions, Conservation Biology, Ecology, Journal of Animal Ecology, Journal of Applied Ecology, Journal of Ecology,* and Oikos. We included all papers that dealt with aliens in any European country. Papers were searched from ISI Web of Knowledge on 22 May 2006 using the words “aliens,” “exotic,” “invasion,” “naturalized,” “non-indigenous,” “nonindigenous,” “non-native,” and “nonnative.” Global papers and models were not included. While other journals include papers on alien species (especially aquatic species), this figure shows an increase in interest in the area based on the ecological and conservation journals examined.

In Europe there has been increasing interest in alien species (Figure 1), but, except for marine systems [7], little effort has been made to integrate information across countries. In 2005, a new European Union consortium called DAISIE (Delivering Alien Invasive Inventories for Europe; http://www.europe-aliens.org) was initiated to address this need across Europe and the Mediterranean Basin for terrestrial, marine, and freshwater environments. DAISIE aims to integrate information on current invasions across Europe through the development of an online, peer-reviewed database of alien species. Linking information on the species' status at both country- and Europe-wide levels should improve understanding and prediction of invasion dynamics [8] and help prevent their spread into new areas.

The research teams in DAISIE were drawn from more than 15 countries in the region by Phil Hulme, who also coordinates this large effort, at National Environment Research Council's Centre for Ecology and Hydrology, in the UK. Representing many of the leading scientists in the field of biological invasions, the group brings together a variety of expertise from academia, government, non-governmental organizations, and private enterprises. In bi-annual workshops, participants address a range of challenging issues. During the latest meeting in February 2006, a central issue for discussion was a “gap” analysis to identify regions in Europe lacking information on the current status and distribution of alien species. To help address these gaps, a European Expertise Registry has been developed (http://www.europe-aliens.org) to identify experts in various countries—at last count, there were 914 experts registered for 1,675 taxa. Since the potential for invasive species control is highest in the early stages of invasion into a new area ([9]; http://www.hear.org/articles/turningthetide/index.html), the database and registry will facilitate the quick assembly of expert teams for an effective response to new invaders.

## Which Are the Worst Alien Species?

An efficient policy dealing with invasive species should raise awareness of the range of impacts caused by different aliens across ecosystems. For this purpose, DAISIE is developing a European invasive alien species information system to provide information on species traits, distribution, and management. A popular awareness-raising strategy is to generate lists of the top (often 100) “worst alien species,” the most high-profile being the global International Union for the Conservation of Nature and Natural Resources (IUCN) list (http://www.issg.org). These lists have been successful at focusing attention on the problem of invasive species, and are widely cited. Following the most recent workshop (after much discussion), DAISIE members drew up a list of 100 species encompassing the breadth of alien taxa, European ecosystems, impacts on biodiversity, ecosystems, human health, and economy. There was limited correspondence with the global IUCN list, highlighting the importance of regional initiatives. The process raised several key questions: Can we directly compare impacts on biodiversity with those on human health? Does the greater visibility of a few vertebrate impacts outweigh less well-understood effects of numerous alien invertebrates? Should we prioritise species that are already a problem or those with large potential to become problematic?

**Figure 2:**
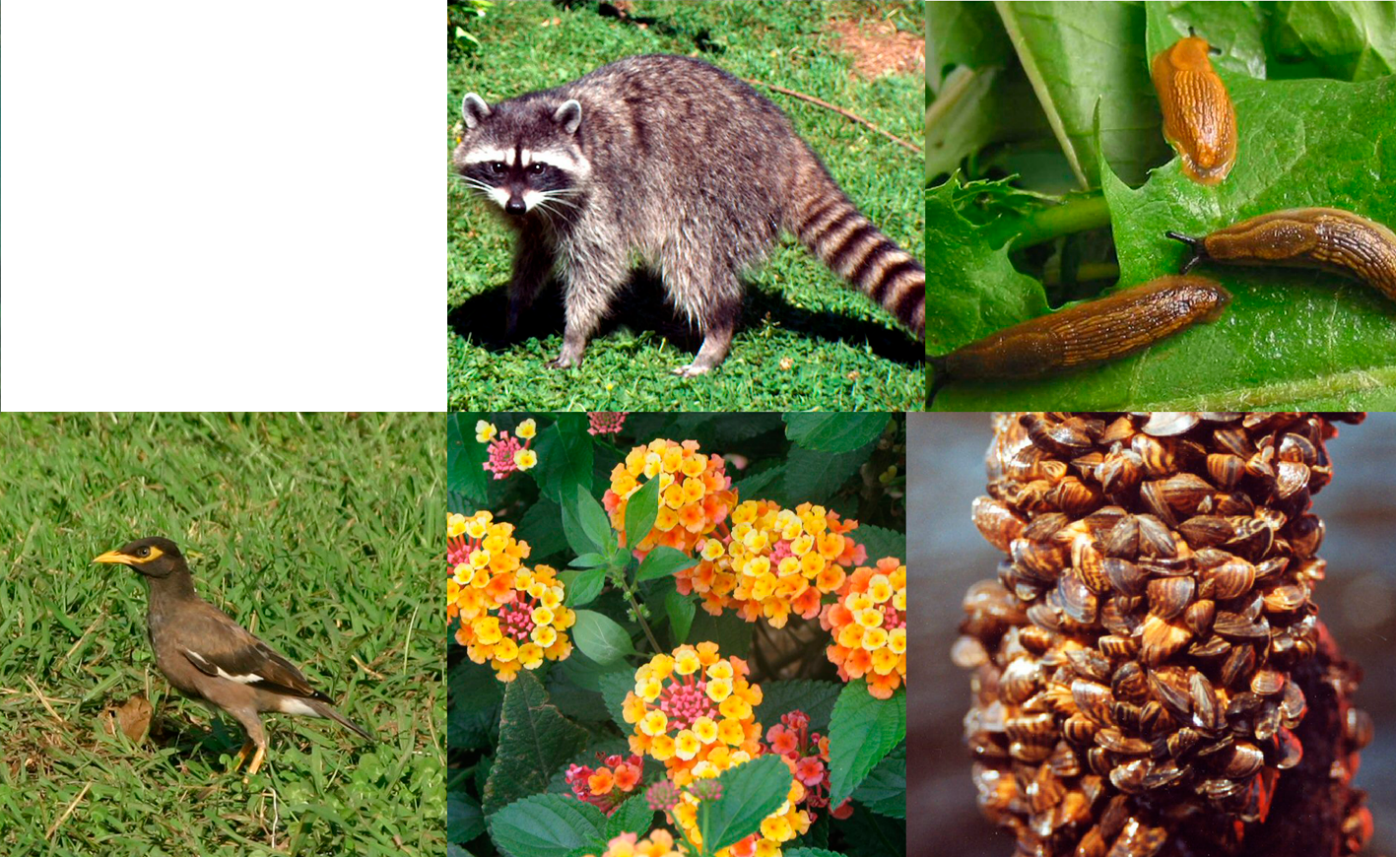
Examples of Alien Species in Europe and the Mediterranean Basin Clockwise from top left: The top left-hand panel has been removed due to copyright restrictions made apparent after publication; the raccoon (Procyon lotor), introduced in Europe in the late 1920s for fur farming, has spread to several central and western European countries, having ecological, agricultural, and health-related impacts; the Spanish slug (Arion vulgaris), originating from the Iberian Peninsula, has invaded many areas of Europe; the zebra mussel (Dreissena polymorpha), introduced from Russia via shipping canals to non-native areas in Europe in the 18th and 19th centuries and later to North America, where it has large negative impacts; the yellow sage (Lantana camara), an invasive weed from tropical America with many cultivars and hybrids, was introduced as an ornamental shrub in Europe and many other areas; and the common myna (Acridotheres tristis), a highly invasive bird species, which was recently introduced to several Mediterranean countries and is rapidly expanding its range. Photos: Raccoon, non-copyright (http://www.sxc.hu/photo/373074), Spanish slug, Inger Weidema; zebra mussel, Dan Minchin; yellow sage, Salit Kark; common myna, Yotam Orchan, Assaf Shwartz.

Even within taxonomic groups, issues arise around criteria for selecting the worst aliens. For some species, a consensus among experts is easily reached; however, in other cases the criteria for selection may yield different lists. For example, the alien Canada goose Branta canadensis is widely distributed across Europe with documented negative effects on agriculture [10], and is often considered one of the worst avian invaders. In contrast, there is less agreement on the impacts of the common myna Acridotheres tristis in Europe (Figure 2). Although currently present in only a few countries in Europe, it is on the IUCN worst-invaders list, being known to rapidly spread in newly invaded areas [11], and to seriously affect breeding success of native birds [12]. The lively discussion generated by these issues highlights a need for robust, clear, and replicable criteria for scientific or policy-making purposes, especially for lists covering wide geographic areas.

## Getting the Word Out

As DAISIE finishes its first year, the alien species database is starting to take form: some of the first results will be reported at the Neobiota conference in September 2006. Ultimately, a European invasive alien species gateway will link the European-wide alien database with the expertise registry and the information system. DAISIE aims to become the European portal of the Global Invasive Species Information Network (http://www.gisinetwork.org). Already, updates submitted by experts across Europe track new alien species, such as a first record in Italy, in March 2006, for the raccoon dog (Nyctereutes procyonoides), a potentially high-impact invader. Together, these databases will provide a powerful online tool to assess impacts of existing biological invasions, and to predict and control future spread. They will be freely available to scientists, decision makers, and the public. This integrated European effort is among the first to address biological invasions at a continental scale, but undoubtedly other large-scale efforts will benefit from this collaboration. A long-term commitment to the maintenance and expansion of this program will be an important challenge to undertake, ensuring the future benefits of information sharing for the preservation of native biodiversity and for society.

## Abbreviations

DAISIE: Delivering Alien Invasive Inventories for Europe
IUCN: International Union for the Conservation of Nature and Natural Resources (The World Conservation Union)
