# Correction: Amassing Efforts against Alien Invasive Species in Europe

**DOI:** 10.1371/journal.pbio.1002090

**Published:** 2015-02-26

**Authors:** The PLOS Biology Staff

## Notice of Republication

This article was republished on January 26, 2015, due to the release of confidential or copyrighted material. A panel in Figure 2 has now been obscured to remove copyrighted material. Please download this article again to view the correct version.
